# Structural aspects of the human small heat shock proteins related to their functional activities

**DOI:** 10.1007/s12192-020-01093-1

**Published:** 2020-04-06

**Authors:** Wilbert C. Boelens

**Affiliations:** Department of Biomolecular Chemistry 284, Institute for Molecules and Materials (IMM), Radboud University, PO Box 9101, NL-6500 HB Nijmegen, The Netherlands

**Keywords:** Small heat shock proteins, α-Crystallin, Oligomerization, Chaperone activity, Protein aggregation

## Abstract

Small heat shock proteins function as chaperones by binding unfolding substrate proteins in an ATP-independent manner to keep them in a folding-competent state and to prevent irreversible aggregation. They play crucial roles in diseases that are characterized by protein aggregation, such as neurodegenerative and neuromuscular diseases, but are also involved in cataract, cancer, and congenital disorders. For this reason, these proteins are interesting therapeutic targets for finding molecules that could affect the chaperone activity or compensate specific mutations. This review will give an overview of the available knowledge on the structural complexity of human small heat shock proteins, which may aid in the search for such therapeutic molecules.

## Introduction

### Human small heat shock proteins

The human genome encodes ten small heat shock proteins (sHSPs), called HSPB1 through HSPB10, some of which are ubiquitously expressed, while others show tissue specificity. They are key components of the cellular protein quality control system, acting as the first line of defense against conditions that affect proteome stability. The defining feature of the sHSP family is a characteristic stretch of 80 amino acid residues, the so-called α-crystallin domain (ACD). This domain is both necessary and sufficient for the formation of dimers, the fundamental building block of the oligomeric structures that often are formed by sHSPs. The ACD is flanked by a less conserved N-terminal domain and a variable C-terminal extension, which both play a crucial role in oligomerization. Human sHSPs have a remarkable degree of structural variation, ranging from dimers (HSPB6, HSPB7, and HSPB8) to heterotetramers with a well-defined subunit ratio (HSPB2/B3) to polydisperse co-assembling oligomeric structures (e.g., HSPB1, HSPB4, and HSPB5). These complexes are able to exchange subunits, which is greatly accelerated by heat or other environmental stresses. This dynamic behavior is a key factor allowing the recognition of client proteins in a specific situation. Here, I discuss the structural aspects of the eight best characterized human sHSPs, HSPB1–8, together with their functional activities.

### Expression of the human sHSPs

**Fig. 1:**
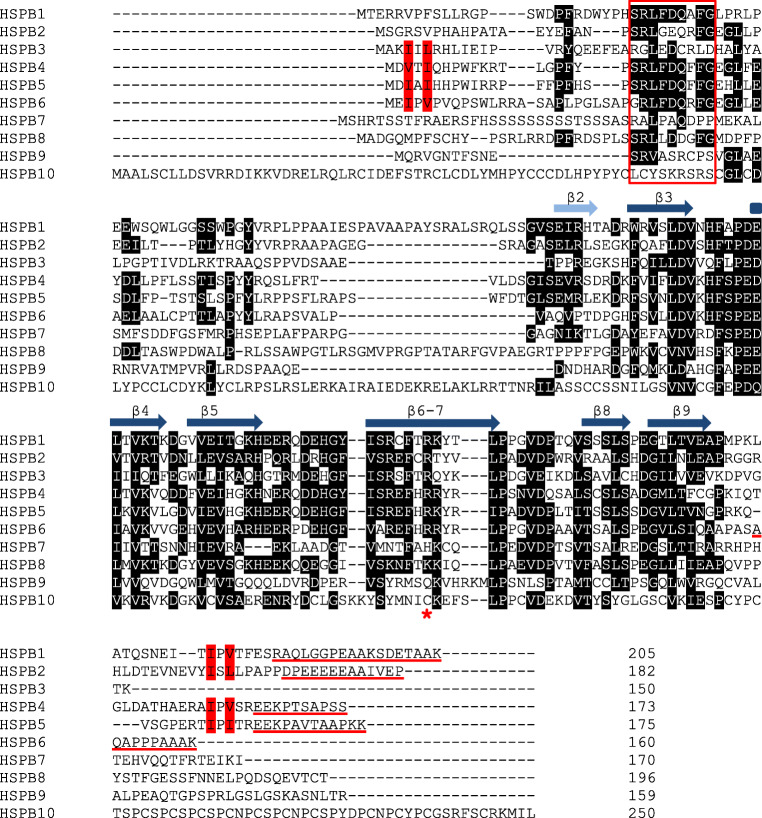
Alignment of the 10 human sHSPs. The ACD is formed by 6 or 7 β-strands, which are indicated by arrowheads above the alignment. The β-strands of the HSPB5 structure are shown (Bagneris et al. 2009). The β2-strand is not always present in the ACD and therefore indicated by a lighter colored arrowhead. The conserved SRLFDQxFG motif in the N-terminal region is marked by a red box. The I/L-X-I/L motif, located in the N- and C-terminal region, is colored red. The underlined sequences at the C-terminus of HSPB1, HSPB2, HSPB4, HSPB5, and HSPB6 highlight the flexible extensions able to tumble freely in solution. The asterisk indicates the position of the conserved arginine, which when mutated is linked to a number of congenital diseases. The alignment is made with Clustal O and manually edited. Residues in black are conserved in 5 or more sHSPs

Already since 1894, HSPB4 and HSPB5 (αA-crystallin and αB-crystallin) are known as structural eye lens proteins, but were only recognized as sHSPs in 1982 based on conspicuous sequence similarities with Drosophila sHSPs (Ingolia and Craig 1982). From that time on, eight more human sHSPs were identified based on their sequence similarities within the conserved 80-residues-long ACD (Table 1) (Fontaine et al. 2003, Kappe et al. 2003). These ten human sHSPs can be considered as paralogous proteins, having originated by gene duplications from a common ancestral gene and are likely to be present in all mammals (Hochberg et al. 2018). Other vertebrates may have distinct subsets of homologous gene sequences (orthologs) and several vertebrates also have additional unique paralogs, indicating that sHSPs are evolutionarily widely diverged (Franck et al. 2004). Alignment of the ten human sHSP sequences immediately highlights the ACD, despite the low sequence identity of around 30% (Fig. 1). Sequence homology outside the conserved domain is even much lower, with the conserved SRLFDQxFG motif in the N-terminal region and the I/L-X-I/L motif in the C-terminal extension as the most prominent exceptions.

**Table 1:** Properties of human sHSPs and disease-associated missense mutations; in bold, the hot spot mutations

Name	Synonyms	Monomeric mass (kDa)	Oligomeric state of isolated protein (Mymrikov et al. 2017)	Main phosphorylation sites	Disease-associated missense mutations (Boncoraglio et al. 2012)
HSPB1	HSP27 HSP25 HSP28	22.8	Large oligomers, size depends on phosphorylation state	S15 S78 S82	G34R; P39L; E42K; G84R; L99M; R127W; S135F; R136W; **R140G**; K141Q; T164A; T180I; P182L; P182S; R188W
HSPB2	MKBP	20.2	Small oligomers, tetramers with HSPB3		
HSPB3		17.0	Dimer/trimer tetramers with HSPB2		R7S; **R116P**
HSPB4	αA-Crystallin	19.9	Large oligomers	S45 S122	R49C; R54P; **R116H**; **R116C**
HSPB5	αB-Crystallin	20.2	Large oligomers, size depends on phosphorylation	S19 S45 S59	P20S; **R120G**; D140N; R157H; G157S
HSPB6	HSP20 p20	16.8	Dimer		
HSPB7	cvHSP	18.6	Dimer		
HSPB8	HSP22 H11 E2IG1	21.6	Monomer/dimer	S24 T87	**K141E**; **K141N**; **K141T**

**Fig. 2:**
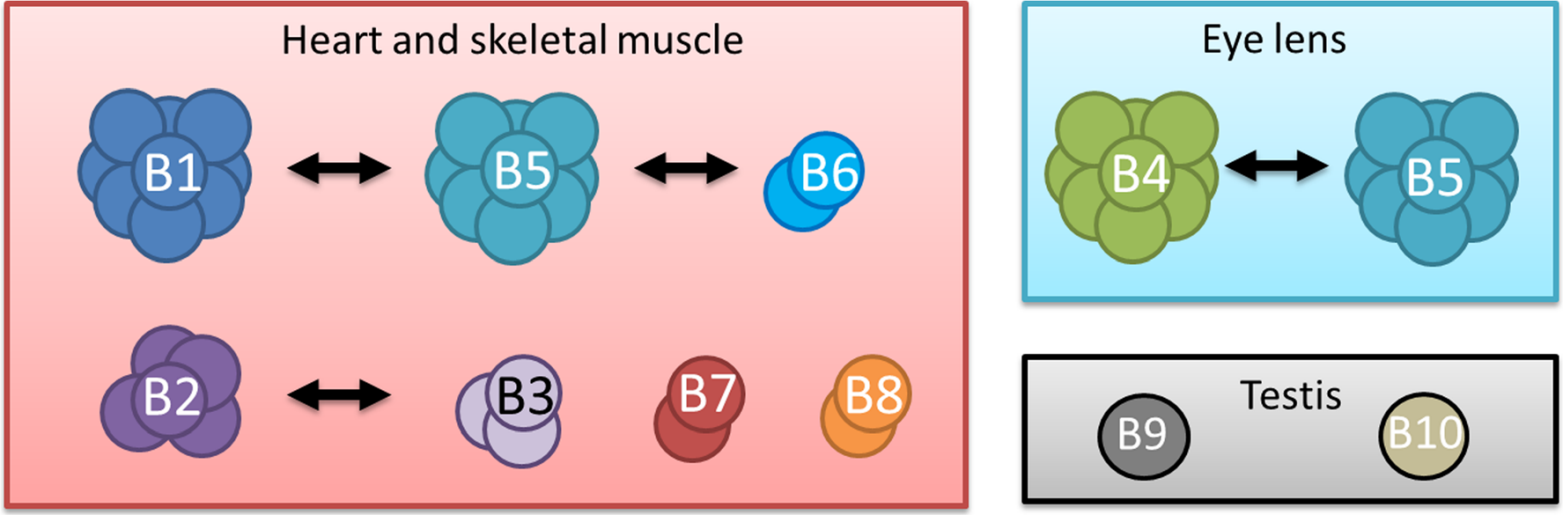
Interactions between the different human sHSPs in muscle, lens, and testis. In heart and skeletal muscle, seven different sHSPs (HSPB1, HSPB2, HSPB3, HSPB5, HSPB6, HSPB7, and HSPB8), in eye lens, two sHSPs (HSPB4 and HSPB5), and in testis, two sHSPs (HSPB9 and HSPB10) are expressed simultaneously at relatively high levels. The arrows indicate which sHSPs have a preference for mutual interactions. The number of subunits is an indication of the size of the complex, except for HSPB9 and HSPB10 of which the complex size is not known yet

The expression of sHSPs is considered to be primarily regulated at the level of transcription (Morrow and Tanguay 2012). They are expressed in a wide variety of tissues, which often contain multiple sHSPs (de Thonel et al. 2012; Verschuure et al. 2003; Xun et al. 2015). Heart and skeletal muscle are the two most noteworthy tissues, in which up to seven sHSPs (HSPB1, HSPB2, HSPB3, HSPB5, HSPB6, HSPB7, and HSPB8) are expressed at the same time and at relatively high levels (Fig. 2). Other tissues express a lower number of sHSPs, mainly HSPB1, HSPB5, HSPB6, and HSPB8. Three sHSP family members are merely limited to specific tissues. HSPB4 is expressed primarily in the lens and together with the highly similar HSPB5 plays an important role in maintaining the transparency of the lens. HSPB9 and HSPB10, the last members assigned to the human sHSP family, are both expressed primarily in the testis (Fig. 2). Because the functional characterization of HSPB9 and HSPB10 is limited, these two proteins will not be discussed further.

The expression levels of sHSPs vary during development. In pig heart, the expression of sHSPs is relatively constant throughout development, whereas the expression in other tissues is transiently upregulated or downregulated (Verschuure et al. 2003). Despite their names, only HSPB1, HSPB5, and HSPB8 are able to respond to a variety of stresses, and the degree by which the expression of these proteins is induced appears to be regulated during development (Zhu et al. 2010). Both the temporal and tissue specificities suggest a changing need for sHSPs, possibly due to variations in sensitivity of tissues for external stimuli.

### The substrates of human sHSPs

sHSPs represent a class of chaperones that bind unfolding substrate proteins in an ATP-independent manner and keep them in a folding-competent state. For the refolding of substrates, the sHSPs transfer the bound substrates to the ATP-dependent HSP70/HSP40 system (Haslbeck and Vierling 2015). This type of chaperone activity enables sHSPs to prevent unfolding proteins from irreversible aggregation, which likely accounts for their role in preventing diseases that are characterized by protein aggregation, such as neurodegenerative and neuromuscular diseases (Carra et al. 2013). The chaperone activity of sHSPs can be determined by temperature- or reduction-induced protein aggregation assays. A comparative analysis of human HSPB1 through HSPB8 showed that HSPB1, HSPB4, and HSPB5 are the most active sHSPs. HSPB2 and HSPB3 have intermediate activity, while HSPB6, HSPB7, and HSPB8 were inactive or showed only moderate activity (Mymrikov et al. 2017). The latter three are the only human sHSPs that do not form large oligomers, which could well relate to their lower chaperone activity (Table 1). Analysis of the sHSP-substrate complexes from heat-stressed cell lysates showed HSPB1, HSPB3, HSPB4, and HSPB5 to be the most promiscuous chaperones able to bind a large number of heat-sensitive proteins, whereas the chaperone activity of the other sHSPs seems to be more substrate dependent (Mymrikov et al. 2017). Intriguingly, HSPB7, which is unable to reduce heat-induced aggregation of cellular proteins, was found to be the most potent member to prevent aggregation of proteins with expanded polyglutamine (polyQ) stretches (Wu et al. 2019). It is likely that HSPB7 interacts with substrates in a very different way compared to the other human sHSPs, since heat-induced aggregates are generally driven by hydrophobic interactions and polyQ aggregation by hydrogen bonding and β-hairpin structures.

The sHSPs do not solely interact with aggregation-prone proteins, based on the findings that a large number of interacting proteins associate under non-stress conditions (Arrigo 2013). For these proteins, it is unknown to what extent their interactions rely on the chaperone activity of sHSPs. The functional activities of these interacting proteins vary from signal transduction, transcription, translation, autophagy, and apoptosis to controlling the cell shape (Arrigo 2013). A well-studied group of interacting proteins are the cytoskeleton proteins, such as tubulin (component of microtubules), actin (component of microfilaments), and vimentin and desmin (components of intermediate filaments). These proteins play an essential role in controlling the cell shape. HSPB1, HSPB4, HSPB5, and HSPB6 have been shown to be able to modulate the assembly and stabilization of one or more of these cytoskeleton proteins, thereby helping to maintain the integrity of the cytoskeleton architecture (Mymrikov et al. 2011; Wettstein et al. 2012). Furthermore, HSPB2 may play a role in controlling the nuclear shape by reorganizing the nuclear lamina, a dense fibrillar network inside the nucleus (Morelli et al. 2017). Another well-studied interactor is the cochaperone BAG3 (BCL-2-associated anthanogene), which physically and functionally links sHSPs with the ATP-dependent HSP70. BAG3 associates with HSPB8, but also with HSPB2, HSPB5, and HSPB6, and acts by facilitating autophagy, thereby preventing misfolded protein accumulation in stressed cells (Rusmini et al. 2017). While several interactors bind multiple sHSPs, some bind very specifically in a highly regulated manner. An example of this is the 14-3-3 protein, which binds only to a phosphorylated form of HSPB6. This interaction triggers smooth muscle relaxation, likely because the binding to HSPB6 displaces binding partners of 14-3-3 (Beall et al. 1999). These examples of interactors of sHSPs under stressed and unstressed conditions demonstrate the multifunctionality associated with this class of chaperones.

## Structure of human sHSPs

### Structure of the conserved α-crystallin domain of human sHSPs

Due to their inherent structural dynamics, sHSPs are extremely difficult molecules for structural studies. In spite of the difficulties, considerable understanding of their tertiary and quaternary structures has emerged in the last years. Although the entire structures remain elusive, various structural details have become available for HSPB1, HSPB2, HSPB3, HSPB4, HSPB5, and HSPB6. Most structural information is available of the conserved ACD as it forms a better defined structure than the more disordered N-terminal domain and C-terminal region. The ACD forms a compact β-sandwich structure with an immunoglobulin-like fold composed of two antiparallel sheets, one of three β-strands (β4-β5-β6+7) and one of which the number of β-strands varies between three and four, dependent on the absence or presence of the β2 strand (β3-β8-β9 or β2-β3-β8-β9, respectively) (Fig. 2). The ACD mediates dimer formation, the interface of which is formed by the antiparallel pairing of the two elongated strands β6+7. This interface generates the extended β-sheet β4-β5-β6+7-β6+7-β5-β4. The interaction between the two sheets is relatively weak with a dissociation constant in the order of a few micromolar (Hilton et al. 2013). Due to this weak interaction, sHSP oligomers can have odd numbers of subunits, containing at least one monomer in addition to the usual dimers (Baldwin et al. 2011a). Recently, it was shown that the unpaired β6+7 strand present in the monomeric form of HSPB1 is not stable and may partially unfold (Alderson et al. 2019). It is possible that this partial unfolding renders the monomer a more potent chaperone.

**Fig. 3:**
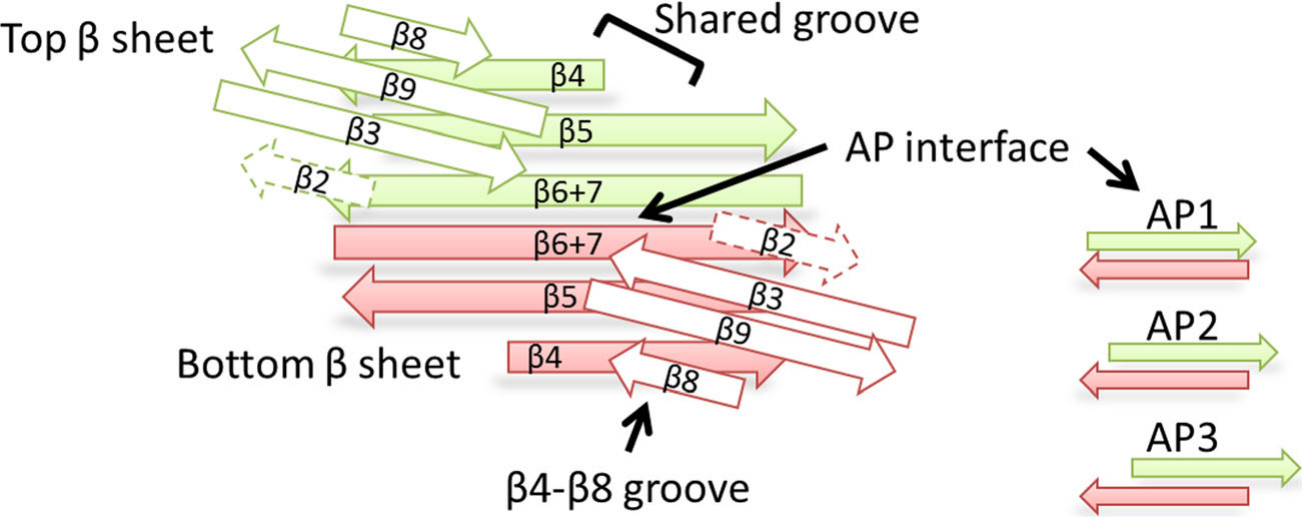
Schematic presentation of the ACD dimer structure. The β-strands of two ACD domains without the connecting loops are shown. These strands form the bottom and top β-sheets of the ACD dimer. The antiparallel β-sheet interface (AP) can vary between three different registers, with the AP1 register having the greatest overlap between the two elongated β-strands and AP3 the least (indicated at the right). The β4-β8 groove at the edge and the shared groove at the dimer interface are indicated. In some sHSPs, the β2-strand is lacking and therefore is shown as dotted arrows

The extended sheet shows considerable flexibility in both crystal and NMR structures of the different sHSPs. First, the antiparallel β-sheet interface (AP) can vary between three different registers, with the AP1 register having the greatest overlap between the two elongated β-strands and AP3 the least (Fig. 3). Most solved dimer structures are in AP2: HSPB1 (3Q9Q) (Baranova et al. 2011), HSPB2/B3 (6F2R) (Clark et al. 2018), HSPB4 (3L1E) (Laganowsky et al. 2010), HSPB5 (2WJ7, 2KLR) (Bagneris et al. 2009; Jehle et al. 2010). The more distantly related HSPB6 has also been found in register AP2 (4JUS (Weeks et al. 2014) and 2WJ5 (Bagneris et al. 2009)). Interestingly, at lower pH, the dimer interface of HSPB5 changes from AP2 (2WJ7, 2KLR) to AP1 register (3L1G) (Laganowsky et al. 2010), suggesting that the interface of HSPB5 is pH sensitive, probably with help of local physiologically titratable histidines. Additional flexibility in the extended sheet is introduced by changes in the curvature of the sheet (Clark et al. 2011). HSPB1, HSPB2/B3, and HSPB4 show a fairly flat structure, but the two monomers of HSPB6 adopt a curved surface, being concave on one side and convex on the other side. The NMR structure of the HSPB5 dimer at pH 7.5 (2KLR) also shows a curved surface, but this curvature might be pH dependent, since the crystal structure at pH 9 (2WJ7) shows a flat structure (Clark et al. 2011; Jehle et al. 2010). Finally, additional flexibility in the extended sheet is introduced by an intrinsic twist, as seen in the solid-state NMR structure of HSPB5 (2KLR) and, to a lesser extent, in the structure of HSPB4 (3N3E and 3L1F) (Laganowsky and Eisenberg 2010). All these variations in dimer structure due to the different ways of introducing flexibility may, in part, give rise to the polydispersity often associated with the full-length proteins.

On the ACD dimer interface, a deep groove is formed that is located between the two β2-β3-β8-β9 sheets and is floored by the extended antiparallel β-sheet (Fig. 3). This shared groove is conserved in the ACD structures of human HSPB2/B3 (6F2R) (Clark et al. 2018), human HSPB5 (2WJ7, 3L1G) (Bagneris et al. 2009; Laganowsky et al. 2010), bovine HSPB4 (3L1F) (Laganowsky et al. 2010), and rat HSPB6 (2WJ5) (Bagneris et al. 2009). The shared groove can be occupied by sequences of the N-terminal region as observed in the crystal structure of phosphorylated human HSPB6 dimer complexed with 14-3-3 dimer and the tetrameric HSPB2-HSPB3 complex (see below). Remarkably, in both complexes, the ACD lacks a β2 strand, thereby creating additional space for the binding of a peptide into the shared groove. Solid-state NMR studies of large assemblies of full-length HSPB5 have shown that the groove can be blocked by curvature of the bottom sheet (Jehle et al. 2010). Thus, it is possible that the ACD dimer is able to fluctuate between open- and closed-groove conformations, thereby affecting the conformation of the N-terminal domain. Furthermore, the open form of the shared groove may serve as a tolerant acceptor site that function as a binding site for substrate proteins that are, during chaperone action, in competition with the N-terminal region.

Besides the shared groove of the ACD dimer, also another groove is present in each ACD. This groove is formed by β4- and β8-strands and is located on the side of the ACD (Fig. 3). This β4-β8 groove plays an important role in the oligomerization by binding the C-terminal I/L-X-I/L motif of a neighboring subunit (see below).

### Structure of the flexible N-terminal domain of human sHSPs

The N-terminal domain (NTD) is the most divergent region among sHSPs, both in length and sequence. The domain is buried in the core of the oligomer, where it is involved in mutual interactions and interactions with the ACDs. Truncation of the NTD yields oligomers that are considerably smaller, indicating that it stabilizes the overall structure of the oligomers (Sudnitsyna et al. 2012). As already mentioned above, the NTD of HSPB1, HSPB4, HSPB5, HSPB6, and HSPB8 contains a conserved SRLFDQxFG motif. Replacing the arginine residue of this motif in each of these sHSPs affects the structure in a different way, suggesting that the motif has distinct roles in the structure (Shatov et al. 2018).

Despite the sequence heterogeneity, the structure of three NTDs has been partially resolved, HSPB5 by solid-state NMR (ssNMR) and HSPB2 and HSPB6 by crystallography. The observed restraints observed for the NTD of HSPB5 by ssNMR are consistent with two helical structures (residues 14–17 and 27–31) in combination with an antiparallel β-hairpin structure (residues 48–50 and 61–63) (Jehle et al. 2011). These structures seem to occur in different conformations, but it is not known whether these structural elements exist simultaneously within a single oligomer or are present in different oligomeric species. The NTDs of HSPB2 and HSPB6 could partially be resolved by crystallization, likely because both form well-defined tetrameric complexes. HSPB2 forms a tetrameric complex with HSPB3 (den Engelsman et al. 2009) and phosphorylated HSPB6 forms a tetrameric complex with 14-3-3 (Sluchanko et al. 2017). By forming these well-defined complexes, the intrinsically disordered NTDs may be transformed into better-defined conformations that allowed obtaining structural information. In the crystal structure of HSPB6, the NTD region from 1 to 38 was traced with the conserved residues 27-RLFDQRFG-34 docking into the shared groove of the ACD dimer (Sluchanko et al. 2017). In the crystal structure of HSPB2/B3 heterotetramer, a long tube of density was observed, which was interpreted as the N-terminal region of HSPB2 containing a hairpin turn 22-SRLGE-26, located within the conserved N-terminal region. The residues 34-LPEEI-38 dock into the shared groove of the ACD dimer of HSPB2, which is a different region compared to that of HSPB6 (Clark et al. 2018). The observed variations in the structure and location of the conserved region might explain why mutating this region can have different effects on the structure of sHSPs (Shatov et al. 2018).

### Structure of the variable C-terminal region of human sHSPs

The C-terminal region is a relative short sequence that varies in length between the different human sHSPs (Fig. 1). The C-terminal regions of HSPB1, HSPB2, HSPB4, HSPB5, and HSPB6 have been shown to contain a highly flexible extension that is typically disordered and tumbles freely in solution (Fig. 1) (Carver et al. 2017). This C-terminal extension presumably acts as a solubilizing agent for the relatively hydrophobic complex that is formed during chaperone action with substrate proteins. The C-terminal regions of HSPB1, HSPB2, HSPB4, and HSPB5 also contain the conserved I/V-X-I/V motif that is typically found in many other sHSPs (Mogk et al. 2019). This motif is able to bind into the groove between strands β4 and β8 of a neighboring ACD and acts as a bridge between the dimers. Because of its flexibility, the C-terminal region can interact with different binding partners independent of their positioning. As a result, the C-terminal region can adopt strikingly different orientations, even within the same oligomer. Because of its dynamic behavior, the C-terminal region may not always be bound to a neighboring subunit and can be entirely detached. The relative populations of these different states likely depend on the conditions and might be modified by posttranslational modifications (Baldwin et al. 2011a). An attractive model is that these C-terminal fluctuations act to regulate access of the β4-β8 groove, which may serve as a binding site for substrate proteins. In this way, the C-terminus could play an important role in regulating chaperone function. HSPB3, HSPB6, and HSPB8 lack the C-terminal I/V-X-I/V motif and probably as a result have a much reduced tendency to form large oligomers. Interestingly, HSPB3 and HSPB6 have an alternative I/V-X-I/V motif in the N-terminal domain (Fig. 1). This N-terminal motif may bind into the β4/β8 groove of the neighboring subunit, thereby stabilizing the dimer structure. Thus, these proteins may use the N-terminal motif to regulate the access of the β4-β8 groove. HSPB4 and HSPB5 also have an N-terminal I/V-X-I/V motif (Fig. 1), but this motif likely does not have the same function due to the presence of the C-terminal I/V-X-I/V motif.

### Homo- and heterooligomerization of human sHSPs

As described above, the sHSP oligomers are formed by multiple labile interactions with the ACD dimer at the core and the flanking regions contributing in a flexible manner. These interactions together determine the total number of subunits that assemble into large oligomers and permit subunit dissociation and re-association (Hochberg and Benesch 2014). Under physiological conditions, sHSP oligomers are relatively stable and show limited subunit exchange. However, in response to stress, the rate of subunit exchange increases dramatically, resulting in alterations in the oligomeric states and distributions. As a result, cryptic modes of substrate interactions are unmasked, thereby diversifying the number of substrate states with which it can interact (Delbecq and Klevit 2019). It is assumed that mainly monomers and not dimers are exchanged between oligomers, based on the finding that disulfide cross-linking of the homodimers at the dimer interphase inhibited heterooligomer formation (Mymrikov et al. 2012).

The formation of heterooligomers is a fascinating aspect of the behavior of sHSPs. This phenomenon was first described for HSPB4 and HSPB5, which are both highly expressed in the lens and form heterooligomers with a 3:1 ratio (Wistow and Piatigorsky 1988). This ratio is mainly determined by the expression level, since in vitro, these two sHSPs form mixed complexes with subunit ratios that reflect the amount of each used (Skouri-Panet et al. 2012). However, the molar ratio of the sHSPs in cells does not completely determine the composition of the heterooligomers that are formed. For instance, it was found that a fraction of HSPB1 (about 10%) was not associated with the heterooligomeric complex formed by HSPB1 and HSPB5, while all HSPB5 oligomeric complexes contained HSPB1 (Arrigo 2013). This indicates that intracellular factors also play a role in determining the composition of the complexes.

Heterooligomerization is believed to occur in many if not all human cell types where multiple sHSPs are expressed. It allows a highly complex and variable adjustment of the chaperone activity and substrate specificity in response to altered conditions. Immunoprecipitation, yeast two-hybrid, and fluorescence resonance energy transfer microscopy data show that most human sHSPs are able to interact with other sHSPs (Fontaine et al. 2005; Sugiyama et al. 2000; Sun et al. 2004). However, several sHSPs show a strong preference for forming specific heterooligomeric complexes (Fig. 2). For example, the three well-studied sHSPs, HSPB1, HspB5, and HspB6, have a strong tendency to form heterooligomeric complexes together that differ from the corresponding homooligomers (Mymrikov et al. 2012). Remarkably, although HSPB6 and HSPB1 both can form homodimers, they have a preference to form heterooligomers mainly consisting of HSPB1/B6 heterodimers. A specific region located in the N-terminal domain of HSPB6 has been identified that dictates heterodimerization (Heirbaut et al. 2017). The preference to form heterodimers reduces the availability of HSPB6 homodimers, and this may influence the sequestration of 14-3-3 (Chernik et al. 2007).

HSPB2 and HSPB3 have a strong preference to interact with each other to form well-defined heterooligomers, consisting of 4, 8, 12, 16, 20, and 24 subunits, each with a subunit ratio of 3:1 (den Engelsman et al. 2009). The HSPB2/B3 heterooligomer has lost its ability to interact with other sHSPs, as is observed for the homooligomer of HSPB2, which can interact with both HSPB6 and HSPB8 (den Engelsman et al. 2009; Sun et al. 2004). The HSPB2/B3 heterooligomer shows poor chaperone and thermoprotective activity and low surface hydrophobicity, while the homooligomeric complexes of HSPB2 and HSPB3 have well detectable chaperone activity (Mymrikov et al. 2017; Prabhu et al. 2012). Remarkably, homooligomeric HSPB2 has the tendency to phase separate, a process that is highly toxic for cells. Thus, a main function of the HSPB2/B3 heterooligomer might be to control the availability HSPB2 (Morelli et al. 2017).

HSPB8 represents an “atypical” member of the sHSP family. It does not form stable complexes with other sHSPs (Datskevich et al. 2012) but instead forms tight complexes with BAG3 in mammalian cells, which is thought to be the obligate partner of HSPB8 (Carra et al. 2008).

HSPB7 is a relatively unexplored member of the family of human sHSPs. Unlike most HSPB family members, HSPB7 does not oligomerize and so far has not been shown to associate with any other member of the sHSP family (Wu et al. 2019).

## Factors that affect the structure of human sHSPs

### Influence of stress conditions on the oligomeric structure of human sHSPs

The oligomeric structures of sHSPs can be affected by various factors, such as temperature, pH, phosphorylation, and other posttranslational modifications. A shift from physiological temperatures to heat stress may require only a few degrees increase to provide sufficient activation energy to increase chaperone activity (Haslbeck et al. 2019). Furthermore, heat stress, and also other stresses, activates stress kinases in cells that influence the oligomeric structures. Of five sHSPs, it is known at which sites they are phosphorylated: HSPB1 at serines 15, 78, and 82; HSPB4 at serines 45 and 122; HspB5 at serines 19, 45, and 59; HSPB6 at serine 16; and HSPB8 at serine 24 and threonine 87 (see Table 1). Most of these sites are located in the N-terminal domain. Phosphorylation increases the overall negative charge, which may affect both the structure and interaction with neighboring protein domains. For HSPB1 and HSPB5, it has been shown that phosphorylation shifts the distribution of the oligomers toward smaller species, likely by destabilization of subunit interfaces (Hayes et al. 2009; Peschek et al. 2013). Remarkably, HSPB1 homooligomers analyzed in cellular extracts show for each phosphoserine a different oligomeric pattern. Phosphoserine 15 was found to be mainly present in small HSPB1 oligomers, phosphoserine 78 in medium-sized oligomers and phosphoserine 82 in the large oligomers (Arrigo 2013). This suggests that each phosphorylation site can have a different impact on oligomer composition.

HspB6 forms dimer structures, which under crowding conditions can associate into larger structures (Sluchanko et al. 2015). These large structures are, like the HSPB1 and HSPB5 oligomers, reduced in size upon phosphorylation. This size reduction may increase the ability of the phosphorylated form of HSPB6 to interact with the 14-3-3 dimer to form a heterotetramer.

Phosphorylation of HspB8 in living cells is achieved by several different kinases (Benndorf et al. 2001). Phosphorylation shifts the equilibrium of HspB8 between monomers and dimers toward dimers. Strikingly, phosphorylation modulates the structure and chaperone-like activity in an unusual way. Mimicking phosphorylation of serine 24, by replacing it by the negatively charged aspartic acid (S24D), reduces chaperone activity of HSPB8, while mimicking phosphorylation at threonine 87 (T87D) has opposite effects on the chaperone properties of HspB8 (Shemetov et al. 2011). Thus, the location of the introduced negative charge in the N-terminal domain differentially affects the chaperone activity.

Another cellular stress that can affect the structure of sHSPs is acidosis, for example, caused by an ischemic stroke (McVicar et al. 2014). Already a decrease in cellular pH over a narrow physiologically range has been shown to destabilize the ACD dimer of HSPB5 (Rajagopal et al. 2015). The weaker dimer interaction correlates with a reduced presence of dimers and increased monomers in the oligomers (Baldwin et al. 2011b). Paradoxically, the weaker interaction between the ACD dimer gives rise to enlarged oligomers, suggesting that the dimer interphase interaction is less crucial for the oligomerization than the N-terminal domain and C-terminal extension. The residue that is responsible for the pH effect is histidine at position 104, which, remarkably, is not localized on the dimer interface (Rajagopal et al. 2015). Mimicking the low pH form of HSPB5 by replacing the histidine by a lysine (H104K) resulted in increased chaperone activity. A pH-dependent, enhanced chaperone activity also has been observed in HSPB1, implying that there may be shared modes of pH-activated chaperone activity (Clouser and Klevit 2017).

### Influence of disease mutations on the structure of sHSPs

Several mutations in sHSPs have been described that are associated with pathologies, such as distal hereditary motor neuropathy (dHMN), Charcot-Marie-Tooth (CTM) disease, and desmin-related myopathy (Boncoraglio et al. 2012). Among these mutations, there are several missense point mutations, as well as mutations leading to frame shifts and the preliminary appearance of a stop codon. Mutations can lead to a gain of function, accompanied by decrease of protein stability and increased tendency for aggregation, or a loss of function, accompanied by inability to form functionally active homo- or heterooligomeric complexes with protein partners and decrease of chaperone activity. One particular residue at a homologous position in sHSPs has been identified as a hot spot for missense mutations. Mutations at this position induce dramatic changes of the structure and functional properties and are linked to a number of congenital diseases. The identified hot spot mutations are R140G in HSPB1 and R116P in HSPB3, which are associated with dHMN; K141E, K141N, and K141T in HSPB8, which is associated with dHMN or CMT; R116C and R116H in HspB4, which are associated with cataract; and R120G in HSPB5, which is associated with cataract, myofibrillar myopathy, and certain forms of cardiomyopathy. This common disease mutation site is situated at the dimer interface on either side of the inside of the shared groove (Bagneris et al. 2009).The conserved arginine (or lysine) residue participates in the formation of salt bridges with negatively charged residues of the neighboring monomer. Mutation of this residue makes the formation of this salt bridge impossible. The crystal structure of the ACD of HSPB5 R120G shows that this mutation causes a major rearrangement of the dimer interface, resulting in the closure of the groove located between the two (β2-)β3-β8-β9 sheets. This rearrangement likely affects the whole structure of the sHSP oligomers leading to a decrease in chaperone activity and modification of interaction with other sHSPs. Noticeably, in cultured cells, the aggregation of the disease-mutated sHSP can be prevented by co-expression of non-mutated sHSPs, likely due to competitive incorporation of its partners into heterooligomeric complexes (Hussein et al. 2015).

Wild-type HSPB8 forms monomers and dimers that weakly interact with other sHSPs. However, when HSPB8 is mutated at common disease mutation site, the protein shows pronounced interaction with wild-type HSPB1 and HSPB5 (Fontaine et al. 2006). This increased propensity of the HSPB8 mutants may result in malfunctioning sHSP complexes. Moreover, the interaction with BAG3 is reduced, which may also participate in the pathogenesis of the neuropathies (Li et al. 2018).

Remarkably, HSPB3 mutated at the disease mutation site has an opposite effect on the interaction with other sHSPs compared to HSPB8. Wild-type HSPB3 forms a stable complex with HSPB2, but the R116P mutation abolishes the interaction with HSPB2. The HSPB3 loss-of-function mutation excludes HSPB2-HSPB3 complex formation and causes aberrant HSPB2 phase separation that likely contributes to the myopathy (Morelli et al. 2017).

Besides the hot spot mutations, several other disease-causing missense mutations have been identified in sHSPs (Table 1). Most of these mutations have been found in HSPB1 and are spread all over the N-terminal domain, crystallin domain, and the C-terminal region, which suggests that there is no other hot spot mutation site present in sHSPs. Most of these mutants are still able to form large stable oligomers in vitro and have a decreased chaperone activity (Nefedova et al. 2015). Since the behavior of sHSPs is rather complicated, it is difficult to predict how these mutations relate to the diseases they cause. Mutations can evoke changes in the oligomeric state of these proteins as well as in their ability to interact with different protein partners.

### Influence of ATP and metal ions on the structure and functioning of human sHSPs

The influence of small molecules on the structure of sHSPs has mainly been focused on HSPB4 and HSPB5 (Biswas et al. 2016). A well-studied molecule is ATP, which has been shown to interact with HSPB4, HSPB5, and the lens-specific HSPB4/B5 heterooligomer, called α-crystallin. The binding site for ATP was experimentally determined to be located in the β4-β8 groove, the region where also the I/L-X-I/L motif interacts (Ghosh et al. 2006). In the presence of ATP, HSPB4/B5 showed a conformational change leading to additional exposure of hydrophobic sites, which was not observed with ADP or AMP. The non-hydrolyzable analogue ATPγS reproduced the effect of ATP, indicating that ATP hydrolysis is not required for the conformational change. ATP enhances the chaperone activity of HSPB4/B5, as observed by measuring aggregation prevention of insulin and refolding of denatured lactate dehydrogenase (Biswas et al. 2016). The high level of ATP present in the eye lens (> 6 mM) thus may have a role in minimizing protein aggregation to maintain proper functioning of the lens.

Also bivalent metal ions have been shown to influence the structure of HSPB4/B5. Of all tested bivalent metal ions, Zn^2+^ appeared to have the strongest effect. Already at a concentration of 1 mM, it can significantly enhance the chaperone activity of HSPB4/B5 (Biswas and Das 2008). Conformational studies revealed that the presence of Zn^2+^ does not alter the secondary and tertiary structures of HSPB4/B5 but increases the hydrophobicity. A mass spectrometric study using diethylpyrocarbonate (DEPC)-modified HSPB4 and HSPB5 indicated that His79, His107, and His115 residues in HSPB4 and His104, His111, and His119 residues in HSPB5 bind to Zn^2+^ (Karmakar and Das 2012). All of these histidine residues are located in the β5-β6+7 strands of the ACD and most likely form with the Zn^2+^ ion an intersubunit bridge to make the dimer structure more stable. Human lens contains about 20 μg zinc per gram dry weight lens tissue (Grahn et al. 2001), but how much of this is actually bound to HSPB4/B5 is not known.

### sHSPs as therapeutic targets

In the last decade, researchers have become increasingly interested in compounds that can serve as therapeutic drugs by influencing the structure and stability of the sHSP complexes. The reason for this increased interest is that much more detailed information became available on the structure and function of the sHSPs in relation with diseases. Besides the involvement of sHSPs in congenital diseases caused by mutations in the genes, also the level of expression of sHSPs has been linked to cataract, several types of cancers, particularly those of carcinoma origin (Arrigo et al. 2007), and neurodegenerative diseases, such as Parkinson’s, Alzheimer’s, and Alexander’s disease and multiple sclerosis (Kampinga and Garrido 2012). sHSPs contain different regions that are suited to dock small molecules, such as the deep groove at the ACD dimer interface and the β4-β8 groove. The interaction of compounds at these regions could affect the speed of subunit exchange and/or the interaction with substrate proteins. The HSPB4/B5 complex has been in focus concerning therapeutic intervention, because of its role in maintaining lens transparency. In search for molecules that bind and stabilize them, the oxysterols lanosterol and 25-hydroxycholesterol have been identified. Both probably bind in the deep groove at the ACD dimer interface. The two compounds are capable of reversing the aggregation of HSPB4/B5 in vitro and partially recovered transparency in animal models of hereditary cataract (Makley et al. 2015; Zhao et al. 2015). Unfortunately, the anti-cataractogenic activity of lanosterol and 25-hydroxycholesterol could not be confirmed by other researchers, suggesting that these compounds might need further chemical adjustments to improve their binding behavior (Daszynski et al. 2019).

Via structure-based molecular docking, a small compound, called NCI-41356, was identified that inhibits the interaction between HSPB5 and vascular endothelial growth factor VEGF165, which plays an important role in the development of breast cancer (Chen et al. 2014). It was found that HSPB5 functions as a molecular chaperone for this growth factor and that disruption of the interaction may downregulate VEGF signaling in breast cancer cells and inhibit proliferation and tumor invasion. An advantage of targeting the interaction with VEGF is that it reduces the chance on possible side effects of directly targeting HSPB5.

Also via structure-based molecular docking, compounds have been identified that interact with HSPB1. The computational drug repositioning approach resulted in several leads, six of which were verified experimentally to interact with HSPB1 and to downregulate its chaperone activity (Heinrich et al. 2016). Since HSPB1 is often overexpressed in cancers that developed resistance against cytotoxic drugs, these HSPB1 inhibitors could improve cancer chemotherapy as a cotreatment together with cytotoxic drugs.

For the treatment of neurodegenerative disorders, a screening of already known antidepressant drugs has been performed to find interactors of HSPB8 (Sehgal et al. 2016). Three compounds were identified that showed binding affinity for HSPB8, and these have to be tested further to determine their potential for the treatment of neurodegenerative disorders.

In conclusion, sHSPs are undoubtedly interesting targets in congenital diseases, cataract, cancer, and neurodegenerative diseases. The available knowledge on the structural complexity of these molecules will certainly aid the search for therapeutic molecules that could neutralize the chaperone activity or compensate specific mutations in these chaperones. However, still much work remains to be done before sHSP drugs can be obtained that could be used in clinical settings without any risk of side effects in patients.
